# Isolation and partial characterization of a highly divergent lineage of hantavirus from the European mole (*Talpa europaea*)

**DOI:** 10.1038/srep21119

**Published:** 2016-02-19

**Authors:** Se Hun Gu, Mukesh Kumar, Beata Sikorska, Janusz Hejduk, Janusz Markowski, Marcin Markowski, Paweł P. Liberski, Richard Yanagihara

**Affiliations:** 1 Departments of Pediatrics and Tropical Medicine, Medical Microbiology and Pharmacology, John A. Burns School of Medicine, University of Hawaii at Manoa, Honolulu, Hawaii, USA; 2 Department of Molecular Pathology and Neuropathology, Faculty of Medicine, Medical University of Łódź, 92-216 Łódź, Poland; 3 Department of Biodiversity Studies, Didactics and Bioeducation, Faculty of Biology and Environmental Protection, University of Łódź, 90-237 Łódź, Poland; 4 Department of Experimental Zoology and Evolutionary Biology, Faculty of Biology and Environmental Protection, University of Łódź, 90-237 Łódź, Poland

## Abstract

Genetically distinct hantaviruses have been identified in five species of fossorial moles (order Eulipotyphla, family Talpidae) from Eurasia and North America. Here, we report the isolation and partial characterization of a highly divergent hantavirus, named Nova virus (NVAV), from lung tissue of a European mole (*Talpa europaea*), captured in central Poland in August 2013. Typical hantavirus-like particles, measuring 80–120 nm in diameter, were found in NVAV-infected Vero E6 cells by transmission electron microscopy. Whole-genome sequences of the isolate, designated NVAV strain Te34, were identical to that amplified from the original lung tissue, and phylogenetic analysis of the full-length L, M and S segments, using maximum-likelihood and Bayesian methods, showed that NVAV was most closely related to hantaviruses harbored by insectivorous bats, consistent with an ancient evolutionary origin. Infant Swiss Webster mice, inoculated with NVAV by the intraperitoneal route, developed weight loss and hyperactivity, beginning at 16 days, followed by hind-limb paralysis and death. High NVAV RNA copies were detected in lung, liver, kidney, spleen and brain by quantitative real-time RT-PCR. Neuropathological examination showed astrocytic and microglial activation and neuronal loss. The first mole-borne hantavirus isolate will facilitate long-overdue studies on its infectivity and pathogenic potential in humans.

A paradigm-disruptive shift in hantavirus host diversity has emerged from the recent discovery of genetically distinct hantaviruses (family *Bunyaviridae*, genus *Hantavirus*) in multiple species of shrews and moles (order Eulipotyphla)^1^,^2^,^3^,^4^,^5^,^6^,^7^,^8^,^9^,^10^,^11^,^12^,^13^,^14^,^15^,^16^,^17^,^18^,^19^ and insectivorous bats (order Chiroptera)^20^,^21^,^22^,^23^,^24^ in widely separated geographic regions in Europe, Asia, Africa and North America. Moreover, phylogenetic analyses, based on partial and full-length hantavirus genomes, suggest that ancestral shrews, moles and/or bats probably predated rodents as the reservoir hosts of primordial hantaviruses^25^,^26^,^27^. However, despite being referred to as novel viruses, nearly all of the 36 newfound non-rodent-borne hantaviruses have not been isolated in cell culture or animals and exist only as viral sequences. As such, almost nothing is known about their biology and transmission dynamics, as well as infectivity and pathogenic potential in humans.

Hantaviruses possess a negative-sense, tripartite RNA genome, designated as large (L), medium (M) and small (S) segments, which encode an RNA-dependent RNA polymerase (RdRp), envelope glycoproteins (Gn and Gc) and a nucleocapsid (N) protein, respectively^28^. Some hantaviruses cause diseases of varying clinical severity, known as hemorrhagic fever with renal syndrome (HFRS) and hantavirus pulmonary syndrome (HPS)^29^. Until recently, rodents (order Rodentia) of the Muridae and Cricetidae families were believed to serve as the exclusive reservoir hosts. The recent flurry of descriptions of newfound non-rodent-borne hantaviruses was presaged by reports, published two to three decades ago which went largely unnoticed, of HFRS antigens in tissues of the Eurasian common shrew (*Sorex araneus*), Eurasian water shrew (*Neomys fodiens*) and European mole (*Talpa europaea*), captured in Russia and the former Yugoslavia^30^,^31^,^32^.

Among the five hantaviruses discovered to date in moles (family Talpidae) from Europe, Asia and North America, Nova virus (NVAV), originally detected in archival liver tissue of a single European mole (*Talpa europaea)* captured in 1999 in Zala County, Hungary^18^, and subsequently shown to be widespread in France^33^ and Poland^34^, is the most divergent^25^,^26^. Here, we report the isolation and partial characterization of NVAV. Since hantaviruses are notoriously difficult to isolate, the long-awaited isolation of NVAV in cell culture is a noteworthy milestone that will accelerate acquisition of new knowledge about its transmission dynamics and facilitate long-overdue studies to ascertain its infectivity and pathogenic potential in humans.

## Results

### Prevalence of NVAV infection

**Figure 1:**
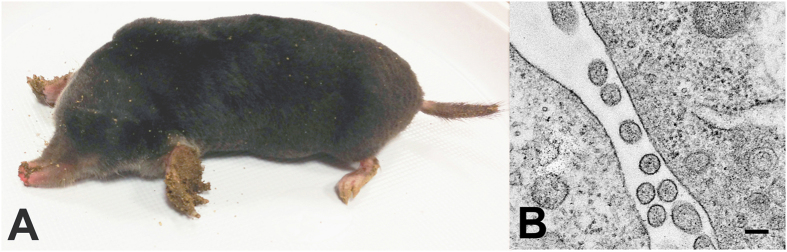
(**A**) European mole (*Talpa europaea*) captured in Huta Dłutowska, in central Poland, in August 2013. (**B**) Thin-section transmission electron micrograph, showing hantavirus particles in Vero E6 cells inoculated with lung homogenate from a NVAV-infected European mole. Bar = 100 nm.

Of 22 European moles (Fig. 1A), captured in Huta Dłutowska between June 21, 2013 and August 26, 2013, NVAV RNA was detected by RT-PCR in lung tissue from 11 (50%). Based on the intensity of the RT-PCR amplicon on ethidium bromide-stained gels, lung tissue homogenates were prepared from four European moles and used for virus isolation attempts.

### Isolation and propagation of NVAV

Initially, NVAV RNA was detected in Vero E6 cells at 17 days following inoculation with a 1% lung tissue homogenate from one of four European moles. Viral RNA was also detected at day 14 of the first passage, but the virus isolate was lost on subsequent passages. After several other failed attempts, NVAV RNA was detected in cells and culture media at 34 days and on subsequent inoculation of fresh Vero E6 cells with culture media, indicating virus replication. At 120 days, the NVAV infectivity in culture media was 8 × 10^3^ plaque-forming units (pfu)/mL. Like all other rodent- and shrew-borne hantaviruses isolated to date, the NVAV isolate did not produce cytopathic effect (or CPE) in Vero E6 cells. Typical hantavirus-like particles, measuring 80–120 nm in diameter, were found by thin-section transmission electron microscopy (Fig. 1B). No other virus-like particles were observed.

### Sequence analysis of NVAV

The full-length S-, M- and L-genomic segments of the cell culture grown NVAV isolate, designated NVAV strain Te34, and the full-length S segment of the NVAV from the original lung tissue, were sequenced. While the overall genomic organization of NVAV was indistinguishable from other hantaviruses, the deduced-amino acid sequence similarities were significantly different from that of rodent-, shrew- and mole-borne hantaviruses, differing by more than 50% for the N protein and Gn/Gc envelope glycoproteins and 40% for the RdRp (Table 1).

The full-length, 1,825-nucleotide S-segment sequence of the NVAV isolate and the NVAV in the original lung tissue were identical, containing a single open reading frame with a predicted N protein of 428 amino acids (nucleotide positions 53 to 1,339), and 52- and 486-nucleotide 5′– and 3′–noncoding regions, respectively. Comparison of the S segment between the NVAV isolate and the prototype NVAV strain MSB95703 from Hungary showed nucleotide and amino acid sequence similarities of 85.6% and 96.5%, respectively. The secondary structure of the predicted N protein of NVAV strain Te34 was virtually identical to that reported earlier for the prototype strain (data not shown).

The entire M-genomic segment of NVAV strain Te34 was 3,590 nucleotides, with a predicted glycoprotein precursor of 1,127 amino acids. The highly conserved WAASA amino acid motif of the M segment was found at amino acid positions 641–645, and the NVAV glycoprotein had six potential N-linked glycosylation sites (five on Gn at amino acid positions 101, 133, 344, 396 and 539, and one on Gc at position 924). The very low degree of amino acid sequence similarity between the GnGc of NVAV and other hantaviruses would predict the absence of antibody cross-neutralization between NVAV and hantaviruses harbored by rodents, shrews, moles and bats (Table 1).

The full-length L-genomic segment of NVAV strain Te34 was 6,563 nucleotides, with a predicted RdRp of 2,157 amino acids, starting at nucleotide position 34 and including 56 nucleotides of the 3′–noncoding region. As with all other hantaviruses, the six conserved motifs (premotif A and motifs A, B, C, D and E) were found. Premotif A had a conserved lysine and two arginine residues. Motifs A, B and D had conserved aspartate, glycine and lysine, respectively. In motif C, there were two conserved aspartic acid residues. The XDD motif, essential for catalytic activity, and motif E, containing the E(F/Y)XS site, were also present. The higher sequence similarity of approximately 60% or more in the L segment probably signified the functional constraints of the RdRp (Table 1).

### Phylogenetic analysis of NVAV

**Figure 2:**
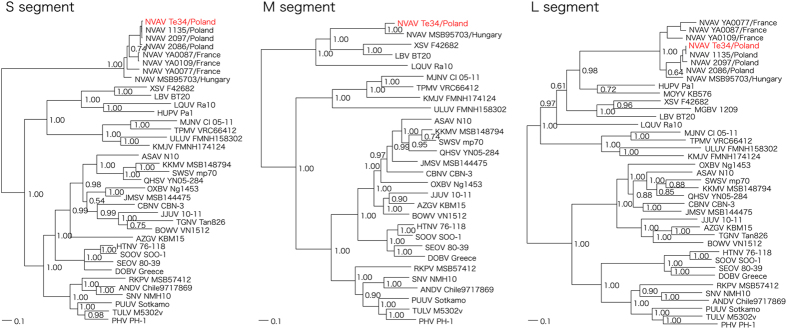
Phylogenetic trees generated by the Bayesian method, using the GTR+I+Γ model of evolution as estimated from the data, based on the alignment of the coding regions of the full- length S, M and L segments of NVAV strain Te34. Topologies of the unrooted phylogenetic trees using the maximum-likelihood method were nearly identical. Relationships are shown to the prototype NVAV strain from Hungary (MSB95703, S: FJ539168; M: HQ840957; L: FJ593498) and to representative NVAV strains from Poland (1135, S: JX990924; L: JX990948; 2086, S: KF515970; L: JX990963; and 2097, S: JX990930; L: KF663727) and France (YA0077, S: KF010573; L: KF010538; YA0087, S: KF010571; L: KF010534; YA0109, S: KF010565; L: KF010521). Other hantaviruses harbored by shrews and moles included Thottapalayam virus (TPMV VRC66412), Imjin virus (MJNV Cl 05-11), Uluguru virus (ULUV FMNH158302), Kilimanjaro virus (KMJV FMNH174124), Asama virus (ASAV N10), Oxbow virus (OXBV Ng1453), Rockport virus (RKPV MSB57412), Jemez Springs virus (JMSV MSB144475), Seewis virus (SWSV mp70), Kenkeme virus (KKMV MSB148794), Qian Hu Shan virus (QHSV YN05-284), Cao Bang virus (CBNV CBN-3), Azagny virus (AZGV KBM15), Tanganya virus (TGNV Tan826), Bowé virus (BOWV VN1512), and Jeju virus (JJUV SH42). Bat-borne hantaviruses included Magboi virus (MGBV MGB1209), Mouyassué virus (MOYV KB576), Huangpi virus (HUPV Pa-1), Longquan virus (LQUV Ra-10), Laibin virus (LBV BT20), and Xuan Son virus (XSV F42682). Also shown are representative rodent-borne hantaviruses, including Hantaan virus (HTNV 76-118), Soochong virus (SOOV SOO-1), Dobrava/Belgrade virus (DOB/BGDV Greece), Seoul virus (SEOV 80-39), Tula virus (TULV M5302v), Puumala virus (PUUV Sotkamo), Prospect Hill virus (PHV PH-1), Sin Nombre virus (SNV NMH10) and Andes virus (ANDV Chile9717869). The numbers at each node are posterior node probabilities based on 150,000 trees. The scale bar indicates nucleotide substitutions per site.

As determined by maximum-likelihood and Bayesian methods, phylogenetic analysis, based on full-length sequences of each genomic segment, demonstrated tree topologies, well supported by bootstrap analysis and posterior node probabilities, showing the NVAV isolate forming a distinctly divergent clade, which comprised other NVAV strains from Hungary, France and Poland (Fig. 2). Similar topologies were found for deduced amino acid sequences of the encoded proteins (data not shown). Collectively, these data strongly supported an ancient hantavirus-host relationship without evidence of host switching.

### Experimental NVAV infection

**Figure 3:**
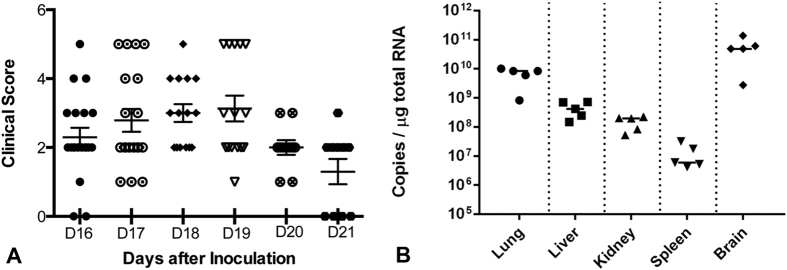
(**A**) Clinical scores of infant Swiss Webster mice inoculated with NVAV strain Te34 by the intraperitoneal route. Data are combined from two independent experiments, consisting of 10 mice each. Mice were monitored twice daily for disease severity. Clinical scores were designated as: 1, ruffled fur/hunched back; 2, hyperactivity/twitching; 3, paresis/difficulty walking; 4, paralysis; and 5, moribund/euthanized. Scores are shown for each mouse, beginning on day 16 post-inoculation. Error bars represent SEM. (**B**) Real-time RT-PCR quantification of NVAV RNA in lung, liver, kidney, spleen and brain of Swiss Webster mice inoculated with NVAV strain Te34. NVAV RNA copies per μg of total RNA are shown for moribund mice sacrificed on day 17 post-inoculation. The solid horizontal line for each tissue signifies the median for five moribund mice.

Of 20 infant Swiss Webster mice, inoculated with 200 pfu of NVAV strain Te34 by the intraperitorneal route, all developed weight loss and hyperactivity, beginning at 16 days. In 11 mice, disease progressed to include hind-limb paralysis, during the ensuing four-day period (Fig. 3A). Moribund mice, with clinical scores of 5, were subsequently euthanized, and their sera had antibodies against NVAV, as determined by the indirect immunofluorescent antibody (IFA) test (data not shown). Using conventional RT-PCR and quantitative real-time RT-PCR, NVAV RNA was detected in lung, liver, kidney, spleen and brain from experimentally infected mice. NVAV RNA copies in the brain were 10- to 100-fold higher than in other tissues (Fig. 3B).

Moreover, brain tissue homogenates from NVAV-infected moribund mice, when inoculated intraperitoneally into new litters of infant mice, produced lethal meningoencephalitis, which was indistinguishable to that observed in infant mice experimentally infected with the NVAV cell-culture isolate. In addition, NVAV sequences amplified from tissues of infected mice were identical to that of the NVAV cell-culture isolate used for experimental infection, as well as to the NVAV originally detected in the European mole lung tissue.

**Figure 4:**
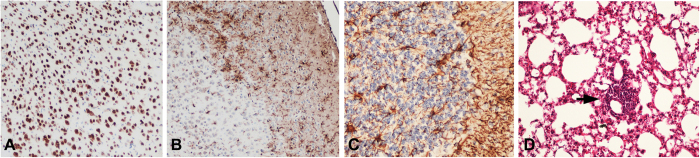
Histopathology in infant mice, 17 days after inoculation by the intraperitoneal route with 200 pfu of NVAV strain Te34. Immunohistochemical staining, showing (**A**) neuronal loss and (**B**) microglial activation in the cerebral cortex and (**C**) widespread astrogliosis in the cerebellum, using primary antibodies against NeuN, Iba1 and glial fibrillary acidic protein, respectively. (**D**) Lung tissue, stained with hematoxylin and eosin, showing perivascular lymphocytic infiltration (arrow). Original magnification: (**A**) and (**B**), X100; (**C**), X200; (**D**), X200.

As assessed by light-microscopic examination of hematoxylin and eosin-stained tissues and by immunohistochemical staining, pathological abnormalities in NVAV-infected infant mice were largely confined to the brain. Perivascular and subpial lymphocytic infiltrates were found, particularly in the cerebellum. Minimal focal loss of neurons was evident in the cerebral cortex, using a neuronal nuclei-specific antibody (NeuN) (Fig. 4A). Microglial activation and astrocytosis, as evidenced by staining for a microglia-specific calcium-binding protein (Iba1) and glial fibrillary acidic protein (GFAP), respectively, were also prominent (Fig. 4B,C). In the lung, perivascular lymphocytic infiltrates were conspicuous with mild widening of the alveolar septa and interstitial infiltration, but without evidence of hyaline membranes or intra-alveolar edema (Fig. 4D).

## Materials and Methods

### Trapping and specimen processing

Moles were trapped using PVC pipes, equipped with flat aluminum latches at both ends, in Huta Dłutowska (N51°35′49.51, E19°22′46.80), near Łódź in central Poland, during June to August 2013. Live-caught moles were euthanized by cervical dislocation, and stored at 4 °C or −20 °C for several hours or days before harvesting of lung tissues, which were stored at −80 °C until used for RT-PCR and virus isolation.

### Ethics statement

Field procedures and protocols, including trapping, euthanasia and tissue processing, were approved by the Łódź Ethical Committee on Animal Testing (14/LB/511/2010 and 29/LB/548/2011) and the Directorate General for Environmental Protection (DOP-OZGiZ.4200/N2732/10/JRO, DOP- OZGiZ.6401.05.25.2011kp.3 and DOP-OZGiZ.6401.05.28.2011kp.1), as well as by the Łódź Regional Directorate for Environmental Protection (WPN-I6631.2010.MS and WPN-L6400.59.2011.MS), in accordance to guidelines of the American Society of Mammalogists^35^. For experimental NVAV infection in Swiss Webster mice, approval was received from the University of Hawaii Institutional Animal Care and Use Committee (protocol 06-055), which adhered to guidelines and regulations of the U.S. Public Health Service Policy on Humane Care and Use of Laboratory Animals and the American Veterinary Medical Association. All experiments involving mice were conducted in consultation with veterinary and animal care staff of the University of Hawaii animal biosafety level-3 laboratory.

### RNA extraction, cDNA synthesis and RT-PCR amplification

As an initial screening for NVAV infection, total RNA was extracted from 20–50 mg of mole lung tissues, using the PureLink Micro-to-Midi total RNA purification kit (Invitrogen, San Diego, CA), and cDNA was synthesized using the SuperScript III First-Strand Synthesis Systems (Invitrogen), then analyzed for NVAV RNA by RT-PCR, using NVAV-specific oligonucleotide primers^33^,^34^.

### Virus isolation and propagation

Attempts to isolate hantavirus from lung tissues of moles with genetic evidence of NVAV infection were made in Vero E6 cells (CRL 1586, American Type Culture Collection, Manassas, VA), using previously described methods^6^. Briefly, subconfluent monolayers of Vero E6 cells, grown in 25-cm^2^ flasks, were inoculated with 1% (w/v) suspensions of lung tissue homogenates, prepared in Dulbecco’s minimum essential medium (DMEM) containing 30% fetal bovine serum, from four NVAV RNA-positive European moles. Cells were sub-cultured at two- to five-week intervals, at which time aliquots of cells and supernatants were examined for NVAV RNA by RT-PCR. Supernatant from NVAV-infected cultures were then used to inoculate fresh Vero E6 cells to demonstrate virus replication and to determine infectivity titers by plaque assay^6^.

### Thin-section electron microscopy

NVAV-infected Vero E6 cells were pelleted and fixed overnight with 2.5% glutaraldehyde (Ted Pella, Inc., Redding, CA) in 0.1 M sodium cacodylate buffer, pH 7.3, washed twice in 0.1 M cacodylate, followed by post-fixation with 1% osmium tetroxide in 0.1 M cacodylate buffer for 1 hour. Infected cells were then dehydrated in a graded series of ethanol washes (30%, 50%, 70%, 85%, 95%, 100%), substituted with propylene oxide, and embedded in LX112 epoxy resin. Ultrathin sections (60 to 80 nm) were obtained using an RMC Powertome ultramicrotome (Boeckler Instruments, Inc., Tuscon, AZ), double stained with uranyl acetate and lead citrate. Grids were viewed with a Hitachi HT7700 high-contrast/high-resolution transmission electron microscope operating at 100kV (Hitachi, Japan), and images were photographed with an AMT XR41 4 megapixel camera (Advanced Microscopy Techniques, Corp., Woburn, MA).

### Whole genome sequencing

Oligonucleotide primers were designed using the MegAlign Clustal W program (DNASTAR Inc., Madison, WI) to obtain the whole genome of NVAV strain Te34 from cDNA prepared from total RNA, extracted from NVAV-infected Vero E6 cells and from lung tissue of the original wild-caught European mole (Table 2). The 5′– and 3′–termini of each segment were amplified using the 3′–Full RACE Core Set (Takara Bio Inc., Otsu, Japan). Nested or hemi-nested PCR was performed in 20 μL reaction mixtures, containing 250 μM dNTP, 2.5 mM MgCl_2_, 1 U of Takara LA Taq polymerase (Takara) and 0.25 μM of each primer (Table 2). Initial denaturation at 94 °C for 2 min was followed by two cycles each of denaturation at 94 °C for 30 sec, two-degree step-down annealing from 46 °C to 38 °C for 40 sec, and elongation at 72 °C for 1 min, then 30 cycles of denaturation at 94 °C for 30 sec, annealing at 42 °C for 40 sec, and elongation at 72 °C for 1 min, in a GeneAmp PCR 9700 thermal cycler (Perkin-Elmer, Waltham, MA)^34^. PCR products were separated, using MobiSpin S-400 spin columns (MoBiTec, Goettingen, Germany), and amplicons were sequenced directly using an ABI Prism 3130 Genetic Analyzer (Applied Biosystems, Foster City, CA)^34^.

### Phylogenetic analysis

To assess the evolutionary relationships, phylogenetic analysis was performed on the coding regions of the full-length S-, M- and L-genomic segment sequences of NVAV and other representative rodent-, shrew-, mole- and bat-borne hantaviruses, using maximum likelihood and Bayesian methods, implemented in PAUP* (Phylogenetic Analysis Using Parsimony, 4.0b10)^36^, RAxML Blackbox webserver^37^ and MrBayes 3.1^38^, under the best-fit GTR+I+Γ model of evolution selected by hierarchical likelihood-ratio test in MrModeltest v2.3^39^ and jModelTest version 0.1^40^. Two replicate Bayesian Metropolis–Hastings Markov Chain Monte Carlo runs, each comprising six chains of 10 million generations sampled every 100 generations with a burn-in of 25,000 (25%), resulted in 150,000 trees overall. Sequences were aligned using Clustal W^41^, and each genomic segment was treated separately in phylogenetic analyses. The posterior node probabilities were based on 2 million generations and estimated sample sizes over 100 (implemented in MrBayes).

### Experimental NVAV infection

To ascertain if experimental NVAV infection resembled that observed with hantaviruses isolated from murid rodents and crocidurine shrews, two-day old Swiss Webster mice were inoculated with 200 pfu of NVAV strain Te34, by the intraperitoneal route. Mice were monitored for weight loss and neurological signs, and the severity of their clinical condition was scored, using a standardized scale^42^,^43^. Terminal sera were tested for antibodies against NVAV by the IFA technique^6^, using NVAV-infected Vero E6 cells spotted onto 10-well slides (Tekdon, Inc., Myakka City, FL) and Alexa Fluor® 488 conjugated goat anti-mouse IgG (H+L) as the secondary antibody (ThermoFisher, Cat. #A-11001, Waltham, MA). Lung, liver, kidney, spleen and brain tissues were collected from moribund mice for histopathology and immunohistochemistry, as well as for quantitation of NVAV RNA by real-time PCR. In addition, brain tissue homogenates from NVAV-infected moribund mice were inoculated intraperitoneally into new litters of infant mice, and NVAV sequences in tissues were compared to that of the NVAV cell-culture isolate and original lung tissue used in the isolation attempts.

### Quantitative Taqman real-time PCR

The primers and probe targeting the NVAV S segment were designed using the Primer Express® software version 3.0 (Applied Biosystems, Foster City, CA). The S-segment forward and reverse primer sequences for real-time PCR were 5′–GCGGTGTTAAGGTCCCAAAA–3′ and 5′–CTTCTGCCTTGATAGCTGATTGAG–3′, respectively, and the probe was 5′–CTTTATGTCTCACTCCCAAC–3′. The probe was labeled with the reporter dye FAM at the 5′–end and quencher dye MGB/non fluorescent at the 3′–end, respectively. Each 20-μL reaction contained 1 μL cDNA, 10 μL 2X TaqMan Gene Expression master mix (Applied Biosystems), 0.5 μL forward and reverse primers (36 μM), 0.5 μL fluorescent probe (10 μM), and 7.5 μL double deionized water. The reaction was performed at 50 °C for 2 min and 90 °C for 10 min, followed by 40 cycles at 95 °C for 15 sec and 60 °C for 1 min, in an ABI 7500 Real-Time PCR system (Applied Biosystems). The standard curve was constructed using RNA from NVAV-infected Vero E6 cells. RNA concentration was measured using a NanoDrop spectrophotometer. After RT-PCR using random primers (10 μM), 10-fold serial dilutions of the cDNA were used in duplicate to generate a standard curve.

## Discussion

Prior to this report, only two non-rodent-borne hantaviruses had been isolated. One is the prototype shrew-borne hantavirus, known as Thottapalayam virus (TPMV), isolated initially in infant mice injected with spleen tissue homogenate from an Asian house shrew (*Suncus murinus*), captured during 1964 in southern India^44^. The other is Imjin virus (MJNV), isolated from the Ussuri white-toothed shrew (*Crocidura lasiura*), captured near the demilitarized zone in the Republic of Korea^6^. This report represents the isolation and partial characterization of the first mole-borne hantavirus, known as NVAV, which is widespread across the geographic distribution of its talpid host, the European mole^33^,^34^.

Because most of the newfound hantaviruses detected in shrews, moles and insectivorous bats have relied on archival tissues, poor tissue preservation, suboptimal RNA quality and limited sample quantity have all conspired to thwart intensive efforts at virus isolation and at acquiring full-length genome sequences. In addition, the high sequence diversity of non-rodent-borne hantaviruses has hampered reliable primer design. Thus, only a few of the hantaviruses harbored by shrews, moles and bats have been fully sequenced, preventing definitive phylogenetic analysis.

The availability of NVAV in cell culture has facilitated the acquisition of its whole genome, resulting in robust phylogenetic trees based on all three segments, with each topology demonstrating that NVAV occupies a highly divergent lineage. NVAV was closely related to bat-borne hantaviruses, suggesting a complex evolutionary history, presumably involving host-switching events, as shown previously for Asama virus in the Japanese shrew mole (*Urotrichus talpoides*)^16^, Oxbow virus in the American shrew mole (*Neurotrichus gibbsii*)^17^ and Rockport virus in the eastern mole (*Scalopus aquaticus*)^19^, as well as periodic episodes of host/pathogen co-divergence through deep evolutionary time^19^,^25^,^26^.

The Gn and Gc glycoproteins, encoded by the M segment, are antigenic and serve as determinants of the protective humoral immune response^45^,^46^. One of the traditional criteria to assign species status to hantaviruses is the absence of antibody cross neutralization, as determined by plaque-reduction neutralization tests (PRNT)^47^. Previously, no antibody cross neutralization could be demonstrated between TPMV or MJNV and rodent-borne hantaviruses^6^,^48^. In the present report, PRNT were not performed because the less than 50% amino acid sequence similarity between the glycoproteins of NVAV and representative hantaviruses for which isolates are available predicted no antibody cross neutralization.

The N-linked glycosylation sites on the Gn (N134, N235, N347, N399 and N609) and Gc (N928) of prototype Hantaan virus (HTNV) strain 76-118 are relatively well conserved among murid rodent-borne hantaviruses and are critical for the conformation of these proteins for transport, receptor binding and antigenicity^49^,^50^,^51^,^52^. Overall, the first, third and fourth N-linked glycosylation sites on the Gn are also generally preserved among cricetid rodent-borne hantaviruses, as well as the few shrew- and mole-borne hantaviruses for which full-length M-segment sequences are available^6^,^10^,^12^,^16^,^17^,^19^,^52^. By contrast, in both NVAV strain Te34, reported here, and NVAV strain BE/Namur/TE/2013/1 from Belgium (GenBank KT004446)^53^, the glycosylation site of N101 on the Gn is unique among all hantaviruses reported to date, including Laibin virus^24^, the only bat-borne hantavirus which has a whole M-segment sequence. To what extent this unique N-linked glycosylation site impacts the function of the Gn is unknown.

As mentioned above, the L-protein motifs of NVAV closely resembled that of other rodent-borne hantaviruses^54^, as well as the six shrew- and one mole-borne hantaviruses for which full-length L segment sequences are available^6^,^7^,^10^,^12^,^14^,^19^. Recently, antibodies directed against the middle portion of the Tula virus L protein were used to study the membrane association of the L protein in infected cells^55^. A similar approach would allow the detection and monitoring of L protein expression in NVAV-infected cells and tissues. These and other studies are now possible with the isolation of NVAV.

The lack of a small animal model has hampered studies aimed at elucidating the pathogenesis of HFRS. Infant mice, as well as certain strains of adult mice (such as C57BL/6, BALB/c, AKR/J, and SJL/J), develop fatal meningoencephalitis following inoculation with HTNV and other HFRS-causing murid rodent-borne hantaviruses^56^,^57^,^58^,^59^,^60^,^61^,^62^, as well as TPMV and MJNV from crocidurine shrews^63^ (Gu *et al.* unpublished observations). Our data indicate that experimental NVAV infection also resulted in neuroinvasive disease. By contrast, infant mice are not susceptible to infection with cricetid rodent-borne hantaviruses, such as Sin Nombre virus (SNV). The absence of convincing human correlates of acute hantaviral neurological disease and of hantavirus antigen and RNA in brains of HFRS and HPS patients argue against the utility and significance of these infant mouse models. That is, the infectivity of hantaviruses in infant and adult mice does not predict the pathogenic potential of hantaviruses in humans.

On the other hand, Syrian hamsters (*Mesocricetus auratus*) may serve as a useful experimental host. Because of their susceptibility to experimental hantavirus infection, Syrian hamsters have been used extensively in the testing of candidate vaccines for HFRS^64^,^65^. Moreover, a lethal HPS model has been developed in adult Syrian hamsters, which recapitulates the clinical and pathological features of HPS in humans^66^,^67^. Recently, we demonstrated that infant and juvenile Syrian hamsters develop a fatal disease, characterized by respiratory distress, weight loss, hind limb paralysis and seizures, following intraperitoneal inoculation with MJNV^68^. High viral loads were found in tissues, and histopathology included hepatic necrosis, widespread inflammation in lungs without pulmonary edema, and extensive MJNV antigens in microvascular endothelial cells. Adult hamsters, on the other hand, were resistant to MJNV infection. Whether or not Syrian hamsters, which are transiently immunosuppressed with dexamethasone and cyclophosphamide, will be rendered susceptible to MJNV infection, as has been shown for SNV, the HPS-causing prototype hantavirus^69^, awaits future study.

Similarly, if NVAV is pathogenic in immunocompetent or immunosuppressed Syrian hamsters, new insights will be gained about hantavirus pathogenesis, and the development of medical countermeasures for hantavirus disease prevention will be accelerated. Alternatively, the development of a chronic NVAV infection model in Syrian hamsters with persistent virus shedding in saliva, urine and/or feces, that presumably resemble natural NVAV infection in European moles, would serve as a convenient model system in which to study NVAV transmission dynamics and genomic reassortment.

Finally, the long-awaited isolation of a highly divergent mole-borne hantavirus will accelerate the acquisition of new knowledge about its infectivity and pathogenic potential in humans. That is, because NVAV infection is highly prevalent in European moles and because European moles often reside near human habitation, clinicians and healthcare personnel should be vigilant for possible NVAV infection in individuals, who develop febrile illnesses or unusual clinical syndromes following known or suspected exposures. The availability of the NVAV isolate will facilitate the development of robust diagnostic tests in search for human infection and disease.

## Additional Information

**Accession codes**: GenBank: NVAV strain Te34 cell-culture isolate: S segment (KR072621); M segment (KR072622); L segment (KR072623); NVAV strain Te34 amplified from lung tissue: S segment (KM403445).

**How to cite this article**: Gu, S. H. *et al.* Isolation and partial characterization of a highly divergent lineage of hantavirus from the European mole (*Talpa europaea*). *Sci. Rep.* **6**, 21119; doi: 10.1038/srep21119 (2016).

## Figures and Tables

**Table 1:** Nucleotide and amino acid sequence similarity (%) between NVAV strain Te34 and other representative rodent-, shrew-, mole- and bat-borne hantaviruses.

Virus strain	S segment		M segment		L segment	
	1,284 nt	428 aa	3,381 nt	1,127 aa	6,471 nt	2,157 aa
NVAV MSB95703	86.4	96.5	82.4	97.7	85.7	96.4
NVAV 1135	99.8	99.5	–	–	99.9	99.8
NVAV 2086	97.7	99.5	–	–	96.3	100.0
NVAV 2097	99.6	100.0	–	–	95.9	99.1
NVAV YA0077	85.0	97.7	–	–	83.5	97.0
NVAV YA0087	86.1	97.4	–	–	85.5	97.4
NVAV YA0109	86.2	97.4	–	–	84.4	97.4
XSV F42682	59.3	56.4	63.2	64.3	71.0	78.8
LBV BT20	57.9	58.3	63.5	58.0	68.8	68.2
LQUV Ra10	54.9	54.6	44.3	44.4	67.9	71.3
HUPV Pa1	61.6	57.6	–	–	71.7	79.8
MOYV KB576	–	–	–	–	70.4	77.8
MGBV 1209	–	–	–	–	68.8	68.6
MJNV Cl 05-11	50.2	44.9	41.2	41.2	65.9	63.0
TPMV VRC66412	49.2	47.0	42.5	42.0	66.2	62.6
ULUV FMNH158302	62.0	48.1	55.9	40.6	64.6	61.4
KMJV FMNH174124	50.8	49.1	49.7	42.1	66.7	62.7
ASAV N10	50.9	48.4	54.6	45.7	66.3	62.1
OXBV Ng1453	51.5	50.0	54.4	45.8	63.9	61.3
JMSV MSB144475	51.4	49.5	60.0	49.8	65.1	62.8
SWSV mp70	51.6	50.7	54.6	60.2	62.8	58.7
QHSV YN05-284	53.0	49.3	61.2	51.1	71.2	73.8
KKMV MSB148794	58.4	48.8	57.3	45.2	64.4	62.3
CBNV CBN-3	57.2	50.2	46.8	44.3	66.5	61.9
JJUV 10-11	50.9	50.7	52.4	45.0	64.6	60.1
TGNV Tan826	64.0	47.6	–	–	70.3	64.2
AZGV KBM15	39.4	50.0	55.6	40.6	65.6	61.4
BOWV VN1512	59.1	49.1	44.9	43.5	64.8	60.6
RKPV MSB57412	57.9	52.3	54.3	48.6	65.4	60.8
HTNV 76-118	50.4	51.4	52.8	45.1	65.7	61.7
SEOV 80-39	57.7	49.5	42.9	45.2	65.3	61.8
SOOV SOO-1	56.6	50.9	50.6	44.9	65.2	61.9
DOBV Greece	57.5	51.9	43.4	45.3	65.0	62.5
ANDV Chile9717869	53.4	51.4	42.9	46.1	64.8	60.7
SNV NMH10	51.3	51.4	43.4	46.6	65.6	61.3
PUUV Sotkamo	58.6	53.3	45.6	46.2	65.3	61.1
TULV M5302v	51.6	51.6	57.2	47.5	65.4	61.4
PHV PH-1	50.1	50.0	59.1	47.1	64.1	60.9

Abbreviations: ANDV, Andes virus; ASAV, Asama virus; AZGV, Azagny virus; BOWV, Bowé virus; CBNV, Cao Bang virus; DOBV, Dobrava virus; HTNV, Hantaan virus; HUPV, Huangpi virus; JMSV, Jemez Spring virus; JJUV, Jeju virus; KKMV, Kenkeme virus; KMJV, Kilimanjaro virus; LBV, Laibin virus; LQUV, Longquan virus; MGBV, Magboi virus; MJNV, Imjin virus; MOYV, Mouyassué virus; NVAV, Nova virus; OXBV, Oxbow virus; PHV, Prospect Hill virus; PUUV, Puumala virus; QHSV, Qian Hu Shan virus; RKPV, Rockport virus; SEOV, Seoul virus; SNV, Sin Nombre virus; SOOV, Soochong virus; SWSV, Seewis virus; TGNV, Tanganya virus; TPMV, Thottapalayam virus; TULV, Tula virus; ULUV, Uluguru virus; XSV, Xuan Son virus. nt, nucleotides; aa, amino acids.– indicates that no sequences are available.

**Table 2:** Oligonucleotide primers for amplification of the full-length S, M and L segments of NVAV strain Te34.

Primer	Sequence (5′ - 3′)	Segment	Polarity
Han-5′end-EcoRI	CTC GAA TTC TAG TAG TAG AC	S/M/L	+
OSM55	TAG TAG TAG ACT CC	S/M/L	+
Shrew-end-EcoRI	CTC GAA TTC TAG TAG T	S/M/L	−
Mole-S732R	GRA AKC CDA TVA CTC CCA T	S	−
Mole-S712R	CAT HAC AGG ACT WAT CA	S	−
Tal-S696F	TGA TNA GYC CTG TNA TGG GAG T	S	+
Tal-S710F	ATG GGA GTG ATA GGC TTT CA	S	+
HTN-S6	AGC TCN GGA TCC ATN TCA TC	S	−
NVAV-S1143F	ACA TGA GAA GGA CTC AGT C	S	+
NVAV-S1169F	ATG CAG ATG GAT CAG CGA	S	+
NVAV-3′endR	TAG TAG TAT ACT CCT TGA AAA GC	S	−
NVAV-M360R	CAC ATA TGC CAA GTA ATG AAC CGT	M	−
NVAV-M330R	AAG TAG TAT CAC CTG CCT GAG TA	M	−
NVAV-M1350R	ACA ATG TCT TTA TCA AGC CGT TGA CA	M	−
NVAV-M1290R	CTA CCA ATA AGA CAT GTT GGA GA	M	−
NVAV-M1440F	ATG TTT CCA TGG ATT GCA CA	M	+
NVAV-M1460F	ATG GCT GTA GAA GTT TGT G	M	+
HTN-M2400R	ARA TAA ANN CCA CAN GCA GTA CA	M	−
HTN-M2355R	CCW GGR CAA TCH VGA GGR TTA CA	M	−
NVAV-M2230F	TAG GTC ATT GGA TGG ATG GAG AGC	M	+
NVAV-M3200F	GAT GGC TTA AAG GCA AGT GCT CCT C	M	+
NVAV-M3260F	CAG ATG CAT TTT CAG ATG GTT CG	M	+
NVAV-L2031R	CAC ATA TAT CTC TAT TGC ACT C	L	−
Bat-L1929F	ATG AAR NTN TGT GCA YTG TTT GA	L	+
Han-L2970R	CCN GGN GAC CAY TTN GTD GCA TC	L	−
Tal-L2759F	ATW GAR GAT TAT TAT GAT GC	L	+
Tal-L2855F	GAA AGG GCA TTN MGA TGG GCN TCA GG	L	+
Han-L2984F	ATG TAT GTN AGT GCW GAT GC	L	+
Han-L3588R	GGN ATH GAN ACN GCA CAN CCY TCA AA	L	−
Han-L-F1	ATG TAY GTB AGT GCW GAT GC	L	+
Han-L-R1	AAC CAD TCW GTY CCR TCA TC	L	−
Han-L-F2	TGC WGA TGC HAC NAA RTG GTC	L	+
Han-L-R2	GCR TCR TCW GAR TGR TGD GCA A	L	−
Shrew-L3370F	GCH CAY CAY TCW GAT GAT GC	L	+
Han-L4330R	TGY TGY TTH GCY TGC AT	L	−
Han-L4297R	ADN GGD GAY TGY AWN GTC AT	L	−
Han-L5239R	TGN AYR CAR TAW GCA TCA TA	L	−
NVAV-L4983F	TGA TGC AGA TGT TAG AGA GTG GTT TC	L	+
NVAV-L5080F	GTT GAT CCT GAA ATA CAA TGT GCT GGC	L	+

Mixed bases: B = G, T, C; D = G, A, T; H = A, T, C; K = G, T; M = A, C; N = A, T, G, C; R = A, G; V = A, G, C; W = A, T; Y = C, T.
